# Use of Enzymatically Converted Cell-Free DNA (cfDNA) Data for Copy Number Variation-Linked Fragmentation Analysis Allows for Early Colorectal Cancer Detection

**DOI:** 10.3390/ijms25063502

**Published:** 2024-03-20

**Authors:** Iva Černoša, Fernando Trincado-Alonso, Pol Canal-Noguer, Kristi Kruusmaa, Alexandre Perera-Lluna

**Affiliations:** 1 Research and Development, Universal Diagnostics d.o.o., 1000 Ljubljana, Slovenia; iva.cernosa@universaldx.com (I.Č.); kristi.kruusmaa@universaldx.com (K.K.); 2 Universal Diagnostics S.A., 41013 Seville, Spain; fernando.trincado@universaldx.com (F.T.-A.); pol.canal@universaldx.com (P.C.-N.); 3 Automatic Control Department, Universitat Politècnica de Catalunya, Av. Diagonal, 647, Les Corts, 08028 Barcelona, Spain; 4 Centre de Recerca en Enginyeria Biomèdica, Universitat Politècnica de Catalunya, Pau Gargallo 5, Av. Diagonal, 647, Les Corts, 08028 Barcelona, Spain; 5 CIBER of Bioengineering, Biomaterials and Nanomedicine (CIBER-BBN), 28029 Madrid, Spain

**Keywords:** cfDNA, fragmentation, colorectal cancer, early cancer detection, WGEM, liquid biopsy

## Abstract

The use of non-invasive liquid biopsy-based cell-free DNA (cfDNA) analysis is an emerging method of cancer detection and intervention. Different analytical methodologies are used to investigate cfDNA characteristics, resulting in costly and long analysis processes needed for combining different data. This study investigates the possibility of using cfDNA data converted for methylation analysis for combining the cfDNA fragment size with copy number variation (CNV) in the context of early colorectal cancer detection. Specifically, we focused on comparing enzymatically and bisulfite-converted data for evaluating cfDNA fragments belonging to chromosome 18. Chromosome 18 is often reported to be deleted in colorectal cancer. We used counts of short and medium cfDNA fragments of chromosome 18 and trained a linear model (LDA) on a set of 2959 regions to predict early-stage (I–IIA) colorectal cancer on an independent test set. In total, 87.5% sensitivity and 92% specificity were obtained on the enzymatically converted libraries. Repeating the same workflow on bisulfite-converted data yielded lower accuracy results with 58.3% sensitivity, implying that enzymatic conversion preserves the cancer fragmentation footprint in whole genome data better than bisulfite conversion. These results could serve as a promising new avenue for the early detection of colorectal cancer using fragmentation and methylation approaches on the same datasets.

## 1. Introduction

Colorectal cancer (CRC) is the third most diagnosed cancer worldwide [1] and the second-leading cause of cancer-related deaths [1]. Despite the continuous progress in screening, as well as improvement in diagnostic and therapeutic methods, early-onset CRC incidence has been rising [2]. Early detection and correct patient stratification are important components for cancer prevention, diagnosis, and successful treatment [3,4]. Currently, CRC is treated by endoscopic and surgical resection, systemic adjuvant chemotherapy, radiation therapy, targeted therapy, and immunotherapy. Since CRC survival is highly dependent upon early diagnosis and treatment, with many patients responding poorly to existing treatments, there is an urgent need for a reliable CRC diagnostic test. Presently, tissue biopsies are the gold standard for tumor identification despite poor patient compliance and difficulties monitoring the disease’s progression through repeated biopsies [5]. The US Preventive Services Task Force (USPSTF) currently recommends screening for all average-risk adults from age 45 to 75 with one of the seven strategies for CRC screening. Strategies differentiate in frequency of testing, scientific approach, cost, invasiveness, and availability. Three of the recommended CRC screening approaches are stool-based tests. They include the fecal immunochemical test (FIT), the high-sensitivity guaiac fecal occult blood test (HSgFOBT), and the multi-target stool DNA (mt-sDNA) test. The next three approaches are direct visualization screening tests, which consist of flexible sigmoidoscopy, computed tomographic (CT) colonography, and traditional colonoscopy. The last strategy is flexible sigmoidoscopy with an FIT, which combines a stool-based test and direct visualization. The USPSTF recommendation for screening for colorectal cancer does not include emerging screening tests such as colon capsule endoscopy (CCE), blood-based screening tests, stool-based microbiome studies, and urinary metabolomics despite potential higher patient compliance [6].

Blood-based screening tests such as rapidly advancing liquid biopsies have opened the doors for the non-invasive detection of cancer [5,7]. The cancer-related features in peripheral blood can be identified by examining the cfDNA fragments [7]. The emerging liquid biopsy methods based on fragment sizes of cfDNA have been previously shown to differentiate between cancer and non-cancer patients [8]. cfDNA in healthy individuals is mostly derived from normal leukocytes and stromal cells, whereas in cancer patients, it can also include tumor-derived DNA, also known as circulating tumor DNA (ctDNA) [5]. DNA fragments floating freely in plasma were first reported in 1948 by Mandel and Metais [9] but have only recently gained traction as a marker for the prediction of response and resistance to targeted therapy and chemotherapy [10,11], analyzing prognosis and tumor burden [12,13], and detecting cancer at early stages [14,15,16].

In recent years, methylation analysis of cfDNA has emerged as a method for early cancer detection [15,16,17]. The most common method for processing samples for methylation analysis includes conversion with Sodium bisulfite (BS). BS chemically modifies unmethylated cytosines, causing their deamination to uracils while retaining methylated cytosines [18]. This enables the detection of the methylation status via sequencing. However, bisulfite conversion also triggers sequencing biases due to a selective and context-specific DNA degradation and incomplete conversion efficiency [19]. Furthermore, bisulfite preparation requires extreme temperatures and extreme pH, which cause the depyrimidination of DNA and generate abasic sites in DNA [20]. Abasic sites cause strand scission and consequently lead to DNA degradation. Therefore, bisulfite treatment can cause DNA backbone breakage upon exposure to heat and alkali due to random base loss at unmethylated cytidines. This major drawback leads to a large decrease in the amount of full-length DNA. To overcome these problems, enzymatic methyl-seq (EM-seq) was recently developed by New England Biolabs as an alternative to the widespread use of BS conversion [21]. EM-seq detects the methylation status using two sets of enzymatic reactions: firstly, TET2 oxidizes methylated cytosines, and secondly, APOBEC3A converts unmethylated cytosines to uracils. This treatment results in a preserved wider range of fragment lengths as compared to BS treatment, which typically results in shorter fragments [18]. Using enzymatic conversion improved the DNA quality and enabled us to apply fragmentomics approaches on libraries made using the EM-seq workflow.

Fragmentomics is a liquid biopsy approach [22,23,24,25] that investigates the landscape of cfDNA fragment profiles in the context of pathological formation and progression. As a methodology, it can also be used to detect copy number variation (CNV) in cancer patients [23,26]. Loss of heterozygosity is known as one of the major types of genetic inactivation in cancer [27], with studies reporting the long arm of chromosome 18 deletion in CRC patients [28], making it an excellent target to be used in a potential diagnostic test. Loss of heterozygosity in chromosome 18 has been reported to be linked to poorer overall 5-year survival for patients with CRC stage III [29]. These findings have been confirmed by Popat et al.’s meta-analysis of data from 27 studies and 2189 patients, which showed that poorer survival was correlated with 18q chromosome deletion [30].

The long arm of chromosome 18, which was previously reported for loss of heterozygosity in sporadic CRC tissues [28] and the deletion of which is postulated to be a biomarker of poor prognosis [29], was selected as the focus of our research since allelic deletions involving chromosome 18q have been reported to occur in more than 70 percent of colorectal cancers [31]. This region includes the SMAD2, SMAD4, and SMAD7 genes coding transducers for receptors of the canonical TGF-β signaling pathway regulating cell development and growth including growth inhibitory signals in the normal intestinal epithelium [32]. Copy number amplifications in these genes could antagonize cell growth arrest and apoptosis [32]. Mutations in SMAD2 and SMAD4 genes have been observed in CRCs [33]; additionally, SMAD4 copy number loss was detected in CRC patients and was associated with tumor progression [34,35], while SMAD7 was shown to be deleted or amplified in CRC [36]. The long arm of chromosome 18 is also the home of a large gene called DCC (deleted in colorectal cancer), which encodes several different protein products as a result of alternative splicing [37], which function as part of a receptor complex for netrin [38], and which is hypothesized to be a tumor suppressor gene [39]. Altogether, the long arm of chromosome 18 has shown high potential for the detection of CRC in the early stages.

Since using whole genome data results in an immense number of features, machine learning techniques are very useful for dimensionality reduction and dealing with the high complexity of datasets. Different approaches combining fragmentomics with machine learning to detect cancer early have been developed so far. For instance, Cristiano et al. extracted the ratio of short and long fragments and applied a gradient boosting model [22]. Other approaches adopted ensemble modeling by combining different classifiers into one. For example, Han et al. combined Support Vector Machines (SVMs), logistic regression (LR), and ridge regression to classify a combination of features from fragmentation profiles and copy number alterations [23], while Jiang et al. used end-motif profile features together with SVMs and LR as classifiers [24]. 

Fragmentation and methylation are the current state-of-the-art areas of interest in early cancer detection using liquid biopsies. Merging both approaches is hindered by the aggressive nature of bisulfite conversion resulting in cfDNA degradation accompanied by a deteriorated cancer signal in the cfDNA fragmentation profile. In the present study, we wanted to investigate the feasibility of using a fragmentation-based approach, combined with CNV analysis, on converted samples, traditionally used for methylation detection. We used samples treated with two different conversion methods and adopted the cfDNA fragment count as a measure of CNV in the regions spanning across chromosome 18 on 72 whole genome enzymatically and bisulfite-converted samples. For estimating the prediction potential of such a method, we used a linear discriminant analysis (LDA) classifier applied to selected features. 

## 2. Results

### 2.1. Overall Approach

cfDNA from the blood plasma of colorectal cancer patients and colonoscopy-confirmed control individuals (CNTs) was used for this study’s purposes. We analyzed cfDNA in the blood plasma of a total of 36 colorectal cancer patients and 36 healthy control individuals (CNTs) described in detail in Section 4. The patient cohort was split into two separate training datasets for enzymatic conversion and bisulfite conversion, respectively, and one common test dataset that consisted of the samples that were processed by both enzymatic and bisulfite conversion. A detailed description of the enzymatic training set, the bisulfite training set, and the test set treated with both conversions can be found under Section 4. CNV knowledge was merged from CRC research together with the number of fragments and fragment length by counting the number of short and medium fragments in the regions of chromosome 18. In total, 83% sensitivity at 92% specificity was achieved on the enzymatically converted test dataset, using regions selected in the training set and the LDA classifier built on it. The bisulfite-converted samples showed a higher level of degradation, compared to the enzymatically converted samples, which also translated into a worse prediction outcome on the test set, with 58.3% sensitivity. Moreover, comparing the general fragment profiles on the three patient samples treated with the enzymatic agent, bisulfite, and no conversion indicated that enzymatic conversion maintains the biological profile of the fragments. 

### 2.2. Enzymatically Converted cfDNA Conserves Biological Fragment Length Better than Bisulfite-Treated cfDNA

In a head-to-head comparison of enzymatic conversion and bisulfite conversion, 24 patient samples were used as part of our test set Three samples used in both the whole genome enzymatic sequencing (WGES) and whole genome bisulfite sequencing (WGBS) sets were also sequenced without conversion using the conversion-free library preparation methodology intended for whole genome sequencing (WGS). 

**Figure 1:**
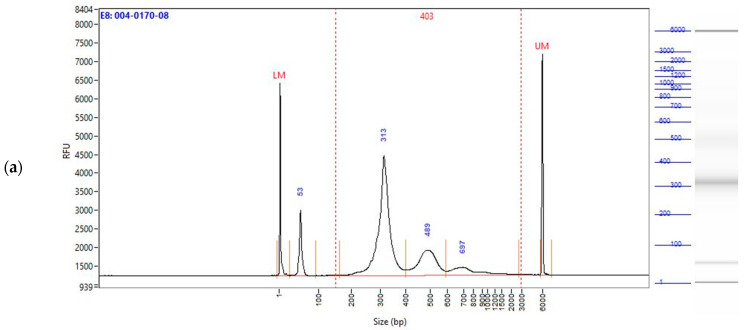

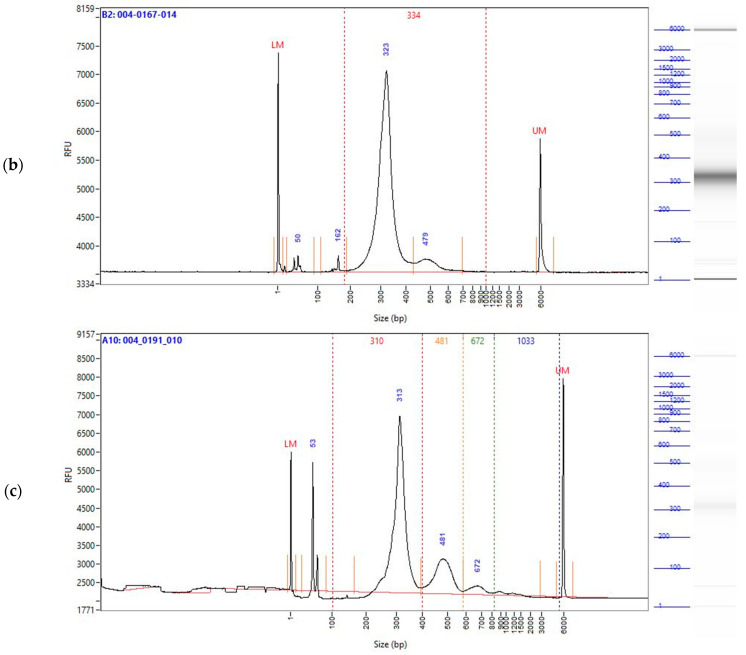
Fragment Analyzer traces, showing fragment sizes measured in bp length, of the 74-year-old, female, CNT sample processed by (**a**) WGEM; (**b**) WGBS; (**c**) WGS method. Numbers on top of each peak for each plot, indicate the average bp length per peak.

**Figure A1:**
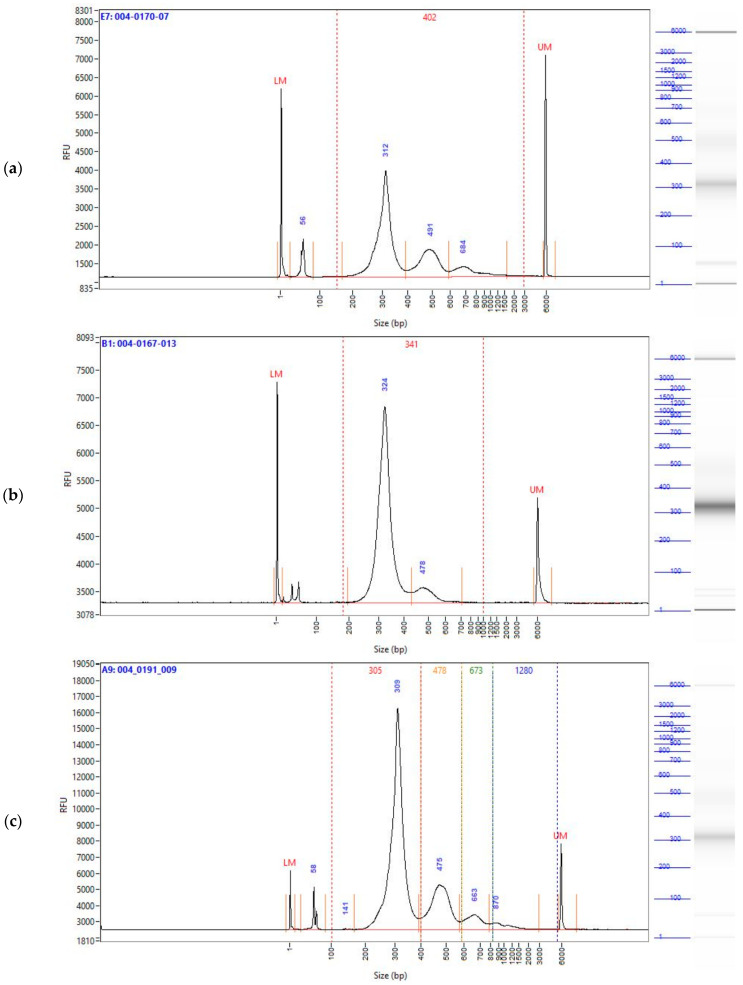
Fragment Analyzer traces of the 70-year-old, male, CRC stage I sample processed by (**a**) WGEM; (**b**) WGBS; (**c**) WGS.

**Figure A2:**
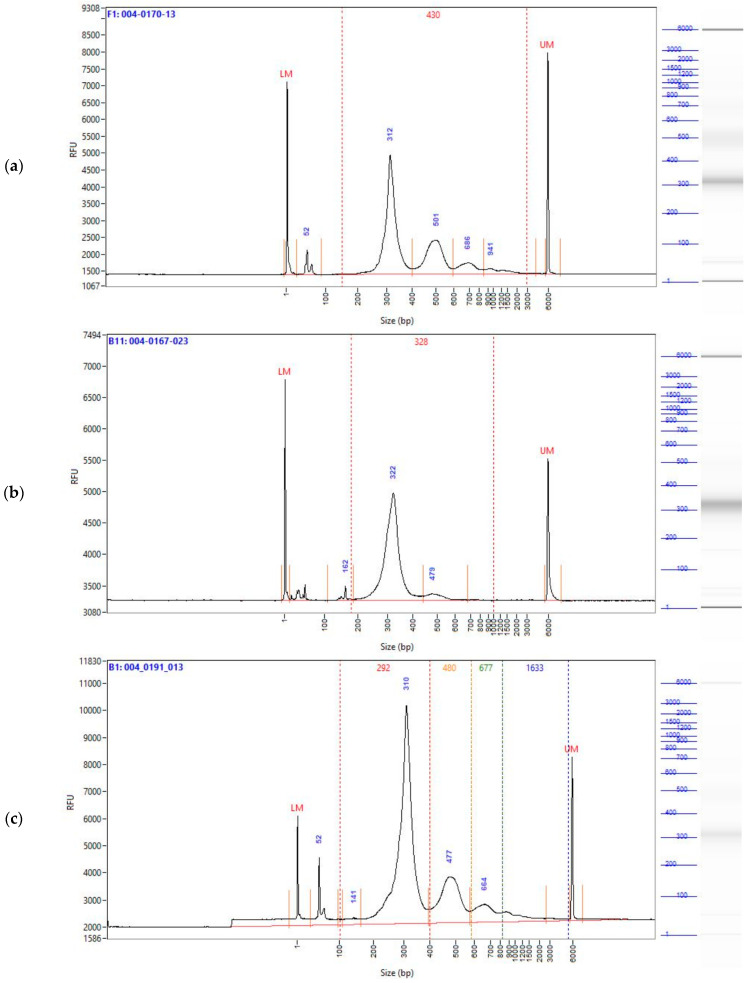
Fragment Analyzer traces of the 52-year-old, male, CNT sample processed by (**a**) WGEM; (**b**) WGBS; (**c**) WGS. Numbers on top of each peak for each plot, indicate the average bp length per peak.

cfDNA for three individual patients was used to prepare the WGES, WGBS, and WGS libraries each. Fragment Analyzer traces were compared per patient in base-pair (bp) size gates pre-set to capture the fragment length of cfDNA treated for library preparation, meaning that the original cfDNA sizes were extended by the addition of adapters and index primers needed for sequencing. Gates were set to capture peaks corresponding to the wrapping over 1, 2, 3, or more nucleosomes: a 100–400 bp interval was selected to capture the maximum range around the fragment size associated with DNA wrapped around one nucleosome, 400–585 bp to capture the fragment peak wrapped around two nucleosomes, 585–825 bp to capture the third peak, corresponding to wrapping over three nucleosomes, and lastly, 825–5500 bp to measure very long fragments. It is important to note that the fragment sizes here represent cfDNA fragments ligated with adapters and index-s for library preparation. As can be seen from the Fragment Analyzer traces (Figure 1a–c, Figure A1a–c and Figure A2a–c), the EM-Seq samples (Figure 1a, Figure A1a and Figure A2a) maintained the biological fragmentation signal from cfDNA much better than the BS samples (Figure 1b, Figure A1b and Figure A2b), when compared to the non-converted WGS data (Figure 1c, Figure A1c and Figure A2c). In the BS data, the longer fragments wrapped around 2–4 nucleosomes were chemically degraded (Figure 1b, Figure A1b and Figure A2b), while in both the EM-seq and WGS data, fragment peaks can be observed in the third and fourth peak.

Table 1 shows the numeric representation of the amount (percentage) of fragments falling into different size gates as output by the Fragment Analyzer for the patient depicted in Figure 1. As can be seen from the table, the WGBS library exhibits a higher percentage of fragments present in the first gates as compared to the WGS and WGES libraries. 

**Figure 2:**
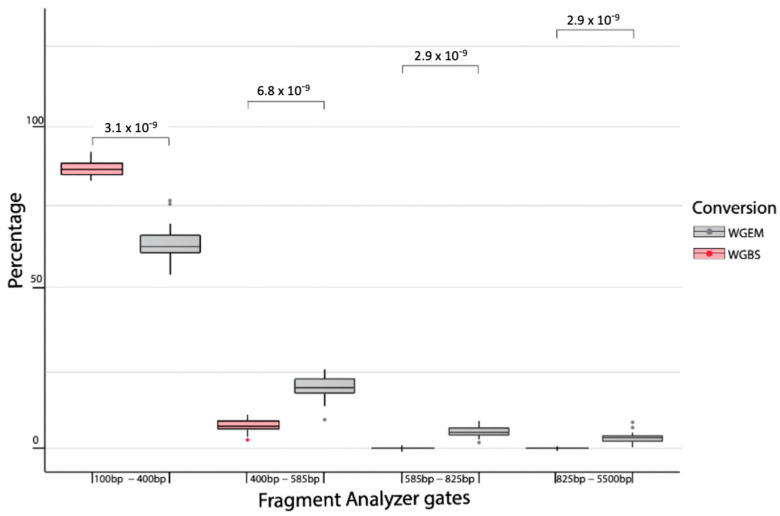
Comparison of percentages of total fragments per base-pair (bp) range, corresponding to nucleosome wrapping, in 24 samples processed by WGBS and WGEM method.

The observations indicated in Table 1 were further confirmed when comparing the percentage of fragments in each pre-set gate for 24 test set samples that were prepared with both the WGES and WGBS treatment (Figure 2). As can be seen from Figure 2, on average, WGBS has a significantly higher % of fragments in gate 1 (100 bp to 400 bp) than WGES, while significantly lower levels in gates 2–4, indicating that degradation of longer fragments might have occurred. 

These findings indicate that enzymatic conversion results in maintaining biological fragment sizes, similar to the non-converted library, as opposed to bisulfite conversion. 

### 2.3. CNV Region Selection

To further evaluate the potential of using enzymatically converted libraries for fragmentomics and fragmentomics-related CNV analysis, 48 patient samples (training set described in Section 4) were treated with WGES and used for evaluating chromosome 18 regions known for the loss of heterozygosity in CRC patients. The patient set was selected to include the majority of the patients with early-stage cancers (75% of the patient set ranging stage 0-IIA), to account for early cancer signals. 

In order to increase accuracy, we decided to check for a correlation between these regions and only select those that were not correlated with each other and as such would provide independent information to the subsequent model building. The 2959 region bins were determined to exhibit a significantly differential signal after filtering for non-correlation criteria. Gene annotation analysis concluded that none of the region bins were located in the SMAD2, SMAD4, SMAD7, or DCC genes but were ranging over centromeres (Chr 18: 17,304,000–20,929,000) instead, indicating a potentially new chromosome 18-related feature being relevant in the CRC context. The selected regions were used as the input data to build an LDA model on the training set.

### 2.4. Detecting Cancer Samples with LDA Model Built over Enzymatically Converted Data

**Figure 3:**
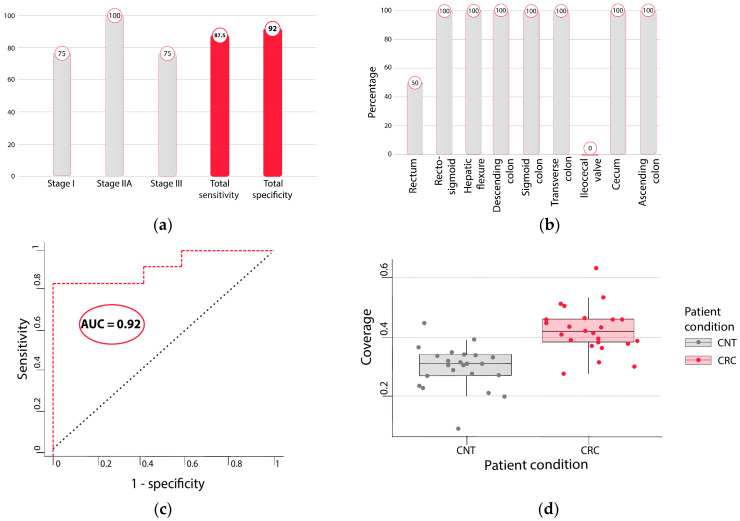
Overview of LDA model performance on enzymatically converted test set: (**a**) sensitivity per stage (%) and total sensitivity (%) and specificity (%); (**b**) sensitivity (%) per CRC location; (**c**) ROC curve of the LDA model; (**d**) box plot of coverage in the most significant region separated by cancer and control samples.

The LDA model built on the training set was applied to the independent enzymatically treated test set (Section 4). The test set included two-thirds of the patients with early-stage cancers (stage I–IIA). The model performance on the test set reached 83% sensitivity (10/12) at 92% specificity (11/12), with stage I sensitivity being as high as 75% (3/4) and stage IIA reaching 100% (4/4) (Figure 3a). Stage III sensitivity was the same as stage I at 75% (3/4). Sensitivity per cancer location was comparable with 83% sensitivity for proximal cancers (5/6) (Figure 3b) and 83% for distal cancers (5/6). All cancers in the rectosigmoid, hepatic flexure, descending colon, sigmoid colon, transverse colon, and cecum were identified and reached 100% sensitivity. One out of two cancers in the rectum was identified, while the ileocecal valve cancer sample was not recognized by our method. The combined sensitivity of early-stages I and II was 87.5%. Specificity was not affected by the presence of benign findings such as hyperplastic polyps, diverticulosis/diverticulitis, or hemorrhoids. The results were independent of age (tested for ages below and above 65) as well as BMI (tested for above and below 30 kg/m^2^). The area under the curve (AUC) of the LDA model on the test set was 0.92 (Figure 3c). Observed coverage in the CRC samples was higher than in the CNT samples, as shown in Figure 3d displaying the most significant region at a *p*-value 3 × 10^−6^ with a Wilcoxon signed-rank test.

### 2.5. Whole Genome Enzymatic Sequencing Performs Better in Cancer Detection than Whole Genome Bisulfite Sequencing

To confirm the hypothesis that sample degradation due to bisulfite treatment interferes with the cancer footprint in cfDNA fragments and would have a potential impact on the early detection of cancer, we repeated the fragmentation-related CNV analysis on the WGBS-treated data.

We used 48 WGBS samples and applied the same feature selection process for chromosome 18 regions, dropping highly correlated features to obtain a similar number of regions as in our original selection with the enzymatic training set. The 48-sample training set was further used for building the LDA model, which was then tested on the same 24 patient samples used in the enzymatic test set. The specificity obtained was comparable to the enzymatically treated samples at 92%. However, the sensitivity was markedly lower at 58.3%. Overall, the AUC of the model was 0.78. This confirms that enzymatic conversion conserves the cancer-related fragmentation signal better than bisulfite conversion, which is especially important for the early detection of cancer, where cancer-related signals are believed to be lower in general. 

### 2.6. Coverage Profiles of Whole Genome Enzymatic Sequencing Are More Similar to Whole Genome Sequencing Profiles than Whole Genome Bisulfite Sequencing Profiles

Bisulfite conversion of DNA introduces pronounced sequencing biases and the differential recovery of the fragments based on their GC content, which impacts genomic coverage in whole genome samples [19]. Unmethylated cytosine-rich sequences are more affected by bisulfite-induced degradation, leading to a significantly higher coverage of cytosine-poor regions and the depletion of genomic regions enriched for unmethylated cytosines.

Since the GC content of our regions was not variable enough for the comparison of degradation in the bisulfite-converted and enzymatically converted to non-converted WGS samples, we computed the GC content in 150 bp long regions of the long arm of chromosome 18 and randomly selected 3000 regions in each of the 5% GC content gates ranging from 15% to 65% GC-content to ensure a wide range of captured sites. Next, we computed the total fragment coverage in the selected 3000 regions in the three biological samples processed by WGS, WGBS, and WGEM.

**Figure 4:**
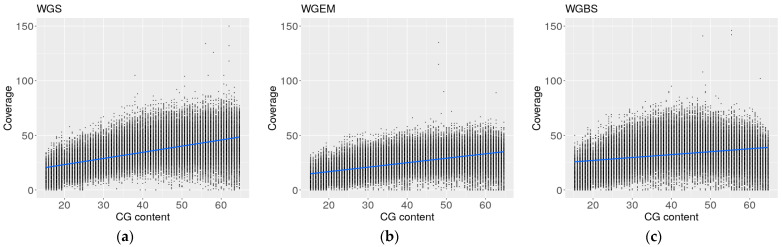
Linear regression of fragment coverage in regions with differential GC content in (**a**) WGS (slope 11.760); (**b**) WGEM (slope 8.521); (**c**) WGBS (slope 21.648).

Linear regression was used to compare the cfDNA coverage profiles in WGS, WGBS and WGEM relative to the GC coverage. The regression coefficient was the highest in the WGS samples with 0.569, followed closely by 0.4106 in WGEM. The WGBS data regression coefficient, with a much lower value at 0.268, was indicative of the depletion of GC-rich cfDNA fragments from the total cfDNA fragment pool (Figure 4). The cytosine-rich DNA fragment degradation was induced by BS conversion and resulted in a skewed representation of cfDNA fragments in the WGBS samples. The WGEM samples did not display this bias and are more similar to the WGS samples (Figure 4).

## 3. Discussion

Colorectal cancer (CRC) remains a leading cause of cancer-related mortality worldwide. Currently, the most commonly used CRC screening tests are the fecal occult blood test with a sensitivity of 12.9%, the fecal immunochemical test with a sensitivity of 73.8%, and colonoscopy—the gold standard reference with a sensitivity of 92.5% [40,41]. Colonoscopy might be the most effective method for CRC screening, but since it is an uncomfortable, invasive, and time-consuming procedure, participation rates are low [42]. This makes a non-invasive liquid biopsy an ideal approach for a test as long as it achieves a high enough sensitivity. In fact, all of the most commonly used CRC screenings suffer from low compliance rates as patients find them either unpleasant to handle or too invasive. This leads to the need for less invasive and more patient-compliant tests, such as liquid biopsy-based tests, which can be easily incorporated into annual physical check-ups for the average-risk population. 

Different methylation-based cfDNA approaches for CRC screening can reach a sensitivity ranging from 48.0% to 90.0% and specificity ranging from 73.0% to 97.0% [40]. A fragmentation-based approach can achieve an accuracy of 0.9 at an area under the curve of 0.94 [43], while an approach combining somatic copy number alteration and fragmentation reached 95% sensitivity at up to 70% specificity [44], showing that non-invasive approaches might soon be comparable to CRC screening using colonoscopy. Challenges, however, persist when aiming for early cancer detection or cancer prevention through pre-cancer location. Pushing early cancer detection further could potentially require a combination of different cfDNA characteristics to capture the rare and heterogeneous signals. For overcoming cost to efficiency issues, new methodologies should be tested where a single workflow can be used to capture different biomarker types. 

We have shown the feasibility of using converted data for fragmentation analysis and the combination of fragmentation and CNV features. Using an LDA algorithm, we detected early stages of CRC in blood plasma with an early-stage (I-II) CRC sensitivity of 87.5% at 92% specificity on an independent test set, surpassing other liquid biopsy-based approaches, especially in early-stage CRC detection. Our data indicate that combining CNV information in chromosome 18 with the cfDNA fragment size information could serve as a promising new avenue for the early detection of colorectal cancer. These results address current unmet clinical needs for a non-invasive and accurate CRC screening test.

Additionally, we have demonstrated that EM-seq preserves libraries much better than bisulfite conversion to the extent that they can be used for the analysis of fragment profiles in cancer detection. This could conceivably open up a new field of cancer detection with methylation and fragmentation approaches used simultaneously in enzymatically converted whole genome samples.

Interestingly, the chromosome 18 features found by our study were not located around previously indicated genes along the long arm of chromosome 18 but rather located around centromeres. Centromere dysfunction, breakage, or compromised centromeric integrity has been reported to be linked to cancer initiation and progression as it leads to chromosomal instability and aneuploidies [45]. 

Additionally, our results were consistent between different colon segments but did indicate a lower performance for the rectum and ileocecal valve. These results could contribute to the sample size but could also indicate the differential signal between the colon, rectum, and ileocecal valve, the latter of which, technically, shares an origin between the small and large intestine. 

However, a couple of potential limitations should be noted. Our analyses were based on CRC-specific CNV markers, limiting the transferability of our approach to other cancer types. To obtain a full picture, this method should be tested on patients with other cancer types with CNV regions appropriate for those types. Another caveat is the small sample size of this study, with an even smaller sample size of early-stage samples, and the fact that the datasets were processed in different batches, potentially resulting in a batch effect. In order to address this, a larger cohort validation of these results is in progress.

## 4. Materials and Methods

### 4.1. Sample Collection

This was a prospective, international, multi-center observational cohort study approved by the CEI de los Hospitales Universitarios Virgen Macarena y Virgen del Rocío with registration Code: 2018/169. We recruited average-risk colorectal cancer patients, either prior to a scheduled colonoscopy as part of a standard colorectal cancer screening program or prior to colonic surgery for primary CRC in several hospitals in Spain, Germany, and Ukraine. Patient cohort was selected to represent patients with early-stage cancers. Patients were informed of the purpose of the study and informed consent was obtained prior to sample collection. Blood samples in volumes of up to 20 mL (4 Streck Cell-Free DNA BCT tubes) were collected from subjects. And were kept at room temperature until plasma isolation, performed within 72 h from collection. Isolated plasma was kept frozen at −80 °C until further processing.

#### Patient and Sample Characteristics

For study purposes, 3 separate patient sets were used as follows: training sets used for enzymatic and bisulfite conversion are described in Table 2 and Table 3. Test set samples that were analyzed with both enzymatic and bisulfite conversion are described in Table 4.

Control and CRC samples both in the enzymatic and bisulfite training set were matched for age and BMI values. In the enzymatic training set, the controls had the equal number of female and male volunteers while there were 2 more female CRC samples than male samples. Gender balance was maintained in the bisulfite training set. With 18 stage 0 to stage IIA samples, 75% of the enzymatic training set samples were comprised of clinically determined early-stage samples (Table 2). In the bisulfite training set, slightly fewer samples were very early stage with 15 stage 0 to stage IIA samples (Table 3). CRC samples in the test set had more males than females, the controls were balanced. Median age and BMI values were also balanced in the test set. Two-thirds of the CRC samples were clinically early-stage I–IIA patients (Table 4).

### 4.2. cfDNA Extraction and Library Preparation

Whole blood samples collected in Streck BCT tubes were processed within 72 h from samples’ collection by a double-spun protocol. The first centrifugation was performed at 1600× *g*/10 min at room temperature. Resulting supernatant was transferred to a new tube and followed by a 2nd centrifugation 5000× *g*/20 min at room temperature. cfDNA extraction was performed using a QIAamp MinElute ccfDNA Midi Kit from Qiagen (Hilden, Germany) using magnetic beads and spin columns that bind DNA, as per the manufacturer’s protocol. An amount of 4–5 milliliters of plasma was used for cfDNA extraction. cfDNA concentration and quality of the extracted samples were measured using fluorescence quantification (Qubit-Invitrogen [Waltham, MA, USA] and/or Varioskan-Thermofisher Scientific [Waltham, MA, USA]) and the profiles were assessed through the capillary electrophoresis technique (5300 Fragment Analyzer System-Agilent Technologies Belgium S.A./N.V. [Zaventem, Belgium]). The lowest measured DNA concentration was 2.03 ng/μL, the highest was 8.54 ng/μL. An amount of 20 ng of cfDNA was used as further input for all the downstream library preparation processes. Artificially methylated and unmethylated spike-ins (Preimium RRBS Kit-Diagenode [Liege, Belgium]) were added to all cfDNA samples prior to library preparation in the conversion workflows using a 10 K-ratio for the evaluation of the conversion efficiency.

### 4.3. Enzymatic Library Preparation

NEBNext^®^ Enzymatic Methyl-seq Kit (New England Biolabs [Ipswich, MA, USA]) was used for library preparation and conversion as per the manufacturer’s protocol. PCR amplification was performed using 4–8 cycles. The optimal number of cycles for library amplification was assessed by qPCR using KAPA SYBR^®^ FAST (Sigma-Aldrich [St. Louis, MO, USA]) on LightCycler^®^ 96 System by Roche (Basel, Switzerland). An amount of 1 µL of post-capture libraries was used for concentration determination using fluorescence quantification (Qubit-Invitrogen and/or Varioskan-Thermofisher Scientific) and 1.5 µL was used for fragment profiles’ assessment using capillary electrophoresis technique (5300 Fragment Analyzer System-Agilent). Six post-conversion libraries were pooled to 4 nM final concentration and were sequenced using a S4 NovaSeq6000 sequencing system (Illumina [San Diego, CA, USA]), with one six-sample pool sequenced per lane, with 2 × 150 bp mode, reaching an average coverage of 37.5×. 

### 4.4. Bisulfite Library Preparation

EZ DNA Methylation-Direct Kit (Zymo Research [Irvin, CA, USA]) was used for conversion following the manufacturer’s protocol. Library preparation was performed with Accel-NGS Methyl-Seq kit (Swift Biosciences [Ann Arbor, MI, USA]), following the manufacturer’s protocol. The optimal number of cycles for library amplification was assessed by qPCR using KAPA SYBR^®^ FAST (Sigma-Aldrich) on LightCycler^®^ 96 System by Roche. An amount of 1 µL of post-capture libraries was used for concentration determination using fluorescence quantification (Qubit-Invitrogen and/or Varioskan-Thermofisher Scientific) and 1.5 µL was used for fragment profiles’ assessment using capillary electrophoresis technique (5300 Fragment Analyzer System-Agilent).

### 4.5. Whole Genome (Non-Converted) Library Preparation

Whole genome libraries were prepared with NEBNext Ultra II DNA Library Preparation Kit (New England Biolabs). The optimal number of cycles for library amplification was assessed by qPCR using KAPA SYBR^®^ FAST (Sigma-Aldrich) on LightCycler^®^ 96 System by Roche. An amount of 1 µL of post-capture libraries was used for concentration determination using fluorescence quantification (Qubit-Invitrogen and/or Varioskan-Thermofisher Scientific) and 1.5 µL was used for fragment profiles’ assessment using the capillary electrophoresis technique (5300 Fragment Analyzer System-Agilent).

### 4.6. Sequencing

Six post-conversion libraries were pooled to 4 nM final concentration and were sequenced using a S4 NovaSeq6000 sequencing system, with one six-sample pool sequenced per lane, with 2 × 150 bp mode, reaching an average coverage of 37.5×. 

### 4.7. Alignment and Raw Data Processing

Raw sequencing files were demultiplexed into FASTQ files. Adapter trimming in raw sequencing reads in FASTQ files was performed using TrimGalore [46]. No additional quality trimming was performed. Reads belonging to the same cfDNA fragment were stitched together before alignment with Bismark. Reads that could not be stitched from the read pair were aligned with Bismark [47] in pair-end mode and fragments were built from read pairs in the output BAM file. Alignment was followed by deduplication and quality filtering. The resulting aligned, deduplicated, and quality-filtered BAM files were used for downstream data analysis. 

### 4.8. Fragment Analyzer Data Processing

Raw data files from 5300 Fragment Analyzer system were processed with ProSize Data Analysis Software version 3 (Agilent). Size gates were pre-set to be 100–400 bp, 400–585 bp, 585–825 bp, and 825–5500 bp for ProSize software to generate percentage outputs per each gate. Output percentages were exported to .csv file for further evaluation.

### 4.9. Data Analysis

All downstream analyses were performed in R 3.6.3 on Linux Ubuntu 20.04.6 LTS using RStudio 3.6.3.

The whole long arm of chromosome 18, as defined in Table 1, was binned in windows of uniform length with the aim of comparing and identifying smaller regions significant for CNV in CRC. The number of short- and mid-range fragments in each bin were counted and used as an input for the LDA model.

### 4.10. Statistical Analysis

Batches of 1000 regions were analyzed in the 48 samples from the training set and tested for correlation. All regions with an average correlation higher than 0.7 were removed to avoid feeding correlated features into the LDA model and to select a smaller number of regions, as well as to reduce the extensive number of possible CRC-specific regions. This left us with 2959 regions, which were tested on 24 samples of the test set not previously seen by the LDA classifier used for CRC identification. Both bisulfite- and enzymatically converted test sets were used. 

## 5. Conclusions

The results of our study indicate that using enzymatically converted data could be used for analyzing CNV-related fragment profiles of cfDNA. Moreover, enzymatically converted data yielded better results than the bisulfite-converted data when applying the CNV fragmentation approach for the detection of early-stage cancers in an independent test set of patients. This opens up a new avenue for the analysis of methylation, fragmentation, and CNV signals from the same analytical workflow, which can be especially important for early cancer detection, where material is scarce and tumor-derived signals are heterogenous.

## Figures and Tables

**Table 1:** Fragment Analyzer output values for percentage of fragments falling into each size gate measured for 74-year-old, female, CNT sample processed by WGEM, WGBS, and WGS method.

Sample	Range	% Total
WGEM	100 bp to 400 bp	60.6
WGEM	400 bp to 585 bp	19.6
WGEM	585 bp to 825 bp	5.9
WGEM	825 bp to 5500 bp	3
WGBS	100 bp to 400 bp	86.8
WGBS	400 bp to 585 bp	9
WGBS	585 bp to 825 bp	1
WGBS	825 bp to 5500 bp	0.5
WGS	100 bp to 400 bp	67.4
WGS	400 bp to 585 bp	19.3
WGS	585 bp to 825 bp	3.9
WGS	825 bp to 5500 bp	1.3

**Table 2:** Training set demographics and characteristics for enzymatic conversion.

Characteristics	Controls	CRC
Age (years, mean (IQR))	68.9 (63–77)	68.9 (63–77)
Female ((n (%))	12 (50%)	13 (54.17%)
Male ((n (%))	12 (50%)	11 (45.83%)
BMI (kg/m^2^, mean (IQR))	28.95 (25–29.3)	29.6 (26.73–30.55)
CRC stage (n)	24	24
Stage 0 (n)		3
Stage I (n)		10
Stage IIA (n)		5
Stage III (n)		3
Stage IIIA (n)		2
Stage IIIB (n)		1

**Table 3:** Training set demographics and characteristics for bisulfite conversion.

Characteristics	Controls	CRC
Age (years, mean (IQR))	68.125 (60.75–75.50)	68.125 (60.75–75.50)
Female ((n (%))	13	13
Male ((n (%))	11	11
BMI (kg/m^2^, mean (IQR))	29.01 (6 NA) (26.275–30.725)	28.58 (7 NA) (25.6–28.7)
CRC stage (n)	24	24
Stage 0 (n)		3
Stage I (n)		6
Stage IIA (n)		6
Stage III (n)		4
Stage IIIA (n)		1
Stage IIIB (n)		1
Stage IV (n)		3

**Table 4:** Test set demographics and characteristics.

Characteristics	Controls	CRC
Age (years, mean (IQR))	65.17 (60.75–70.75)	64.4 (61–69.5)
Female ((n (%))	6 (50%)	5 (41.67%)
Male ((n (%))	6 (50%)	7 (58.33%)
BMI (kg/m^2^, mean (IQR))	28.2 (24.95–30.85)	28.92 (26.37–31.3)
CRC stage (n)	12	12
Stage I (n)		4
Stage IIA (n)		4
Stage III (n)		4

Three patients, for whom the libraries were prepared with WGES, WGBS, and WGS methods were as follows: 74-year-old, female CNT; 70-year-old, male, CRC stage I; 52-year-old male, CNT.

## Data Availability

The data presented in this study are available on request from the corresponding author (accurately indicate status).
